# Risk factors associated with frequent infections in the elderly

**DOI:** 10.1186/1939-4551-8-S1-A57

**Published:** 2015-04-08

**Authors:** Marek L Kowalski, Joanna Makowska, Aleksandra Wardzynska

**Affiliations:** 1 Medical University of Lodz, Poland

## Background

Aging is associated with structural and functional changes in the immune system which may be responsible for a higher incidence of infection in the elderly.

The aim of the study was to assess the prevalence of infections among elderly students of the Academy of Healthy Ageing and to establish factors associated with frequent infections.

## Methods

The questionnaire including questions about infections, medication, comorbidities and socio-economic background was filled by 157 students (83% women and 17% men, mean age 68.2) of Academy of Healthy Ageing, one of the initiatives of the Healthy Ageing Research Centre. Univariate and multivariate logistic regression analysis was used to establish factors associated with frequent respiratory, urinary and cutaneous infections.

## Results

At least one infections of respiratory system during last year were reported by 71% of elderly students. The mean number of infections was 3.2 per patient. Herpes simplex infection was reported by 40% of students (mean number 0.9) and 32% study participants had urinary tract infections (mean number 0.74). Frequent respiratory, urinary and cutaneous infections were reported by of 45.2%, 10.2% and 14% of students respectively. 37% of elderly were treated with antibiotics at least once during past year, and only 2% of study subjects were hospitalized due to infection.

The risk factors for frequent (defined as more than 3 per year) respiratory infections in multivariate analysis were inflammatory arthritides (RA, gout) OR=1.64 (CI95% 1.01-2.67) and polytherapy (more than 5 prescription drugs) (OR=1.93 (CI95% 1.11-3.36). Multivariate analysis did not reveal the risk factors for frequent infections of urinary tract or Herpes simplex infection. In univariate analysis frequent urinary tract infections were associated with diabetes (OR=2.32 (CI95% 1.26-4.27)) and anti-diabetic treatment (OR=2.35(CI95%1.21-4.6)).

## Conclusions

Frequent infections among elderly students of the Academy of Healthy Ageing are associated with certain comorbidities and medications used.

